# Imported Cholera Cases, South Africa, 2023 

**DOI:** 10.3201/eid2908.230750

**Published:** 2023-08

**Authors:** Anthony M. Smith, Phuti Sekwadi, Linda K. Erasmus, Christine C. Lee, Steven G. Stroika, Sinenhlanhla Ndzabandzaba, Vinitha Alex, Jeremy Nel, Elisabeth Njamkepo, Juno Thomas, François-Xavier Weill

**Affiliations:** University of Pretoria, Pretoria, South Africa (A.M. Smith);; National Institute for Communicable Diseases, Johannesburg, South Africa (A.M. Smith, P. Sekwadi, L.K. Erasmus, J. Thomas);; US Centers for Disease Control and Prevention, Atlanta, Georgia, USA (C.C. Lee, S.G. Stroika);; National Heath Laboratory Service, Johannesburg (S. Ndzabandzaba, V. Alex);; University of the Witwatersrand, Johannesburg (S. Ndzabandzaba, V. Alex, J. Nel);; Institut Pasteur, Université Paris Cité, Paris, France (E. Njamkepo, F.-X. Weill)

**Keywords:** cholera, Vibrio cholerae, Vibrio cholerae serogroup O1, sequence type 69, bacteria, enteric infections, South Africa, Malawi

## Abstract

Since February 2022, Malawi has experienced a cholera outbreak of >54,000 cases. We investigated 6 cases in South Africa and found that isolates linked to the outbreak were *Vibrio cholerae* O1 serotype Ogawa from seventh pandemic El Tor sublineage AFR15, indicating a new introduction of cholera into Africa from south Asia.

The seventh cholera pandemic arrived in Africa during 1970, and the related cholera strain, *Vibrio cholerae* O1 biotype El Tor (7PET), has since become endemic in many countries in Africa (*1**–**3*). As of March 20, 2023, at least 24 countries globally reported ongoing cholera cases. Several countries in southeastern Africa, in particular Malawi and Mozambique, were experiencing outbreaks. In addition, outbreaks were spreading regionally, including to Tanzania, Zambia, Zimbabwe, and South Africa. The largest active cholera outbreak on the continent was in Malawi: 54,841 cases and 1,684 deaths reported during February 28, 2022–March 20, 2023 (*4*). 

South Africa is not considered endemic for cholera; previous outbreaks have typically been associated with importation events. However, cholera remains under active surveillance in South Africa. The National Institute for Communicable Diseases is notified of all suspected cases. All *V. cholerae* isolates are submitted to the Centre for Enteric Diseases, which provides further laboratory investigation, including phenotypic and genotypic characterization (Appendix 1) (*5*). Ethics approval was obtained from the Human Research Ethics Committee, University of the Witwatersrand, Johannesburg, South Africa (protocol reference no. M210752). 

As of February 28, 2023, a total of 6 cholera cases in South Africa had been laboratory confirmed by the Centre for Enteric Diseases; fecal samples were collected from patients February 1–23, 2023. All cases occurred in Gauteng Province (Table); 3 case-patients were female (19–44 years of age) and 3 male (23–41 years of age). Cases 1–3 were imported or import-related cases. Case-patients 1 and 2 (sisters) left Johannesburg on January 15, 2023, and traveled together to Chinsapo, Lilongwe, Malawi, in one of the districts reporting active outbreaks, where they stayed until their departure on January 29, 2023. Both women reported onset of symptoms within 12 hours of departure during the bus trip back to Johannesburg. Case-patient 3 was a close household contact of case-patient 1. Case-patients 4–6 acquired infection locally and were classified as indigenous cases; none had travelled or had any link to the imported or import-related cases or to one another. We identified isolates associated with all 6 cases as *V. cholerae* O1 serotype Ogawa and all were PCR-positive for the cholera toxin–producing gene. 

**Table:** Characteristics of cholera cases and classification of *Vibrio cholerae* O1 serotype Ogawa sequence type 69 isolates from patient fecal samples, Gauteng Province, South Africa, 2023

Case	Date sample collected	Cholera case classification	Comment on case classification	Patient age, y/sex	Clinical manifestations
1	2023 Feb 1	Imported case	Infected in Malawi	37/F	Acute diarrhea and dehydration
2	2023 Feb 2	Imported case	Infected in Malawi	44/F	Mild diarrhea
3	2023 Feb 5	Related to imported case	Close household contact of case-patient 1 (direct link to imported case)	41/M	Acute diarrhea and dehydration
4	2023 Feb 16	Locally acquired indigenous case	No travel history; no evidence of direct link to an imported case	27/M	Acute diarrhea and dehydration
5	2023 Feb 12	Locally acquired indigenous case	No travel history; no evidence of direct link to an imported case	23/M	Mild diarrhea
6	2023 Feb 23	Locally acquired indigenous case	No travel history; no evidence of direct link to an imported case	19/F	Mild diarrhea

We used whole-genome sequencing, comparative genomics, and phylogenetic analysis to further characterize the isolates (Appendix 2 Tables 1, 2). The 6 *V. cholerae* O1 isolates had similar genomic features, including the toxin-coregulated pilus gene subunit A gene variant, *tcpA*^CIRS101^, a deletion (ΔVC_0495-VC_0512) within the vibrio seventh pandemic island II (VSP-II), and an SXT/R391 integrating conjugating element called ICE*Vch*Ind5, encoding resistance to streptomycin (*strAB*), sulfonamides (*sul2*), trimethoprim (*dfrA1*), and trimethoprim/sulfamethoxazole (*dfrA1* and *sul2*) and resistance or intermediate resistance to chloramphenicol (*floR*). The isolates also had mutations of *VC_0715* (resulting in the R169C substitution) and *VC_A0637* (resulting in the premature stop codon Q5Stop) conferring nitrofuran resistance, and of the DNA gyrase, *gyrA* (S83I), and topoisomerase IV, *parC* (S85L) genes, conferring resistance to nalidixic acid and decreased susceptibility to ciprofloxacin (*3*,*6*). The isolates also had a specific nonsynonymous single-nucleotide variant (SNV) in the *vprA* gene (*VC_1320*) (resulting in the D89N substitution), conferring susceptibility to polymyxins (*6*). 

To place these 6 isolates into a global phylogenetic context, we constructed a maximum-likelihood phylogeny of 1,443 genomes (Appendix 2 Table 3) with 10,679 SNVs evenly distributed over the nonrepetitive, nonrecombinant core genome. All isolates from South Africa clustered together (median pairwise distance of 4 [range 0–8] core-genome SNVs) in the 7PET lineage wave 3 clade, containing isolates carrying the *ctxB7* allele (Figure) (*6*). However, those isolates did not belong to any of the sublineages previously found in Africa (AFR1 and AFR3–AFR 14) (Figure) (*3*,*6*,*7*); instead, they tightly grouped with genomes of south Asia variants, suggesting that the 2022–2023 cholera outbreak in Malawi and cases in South Africa in our study were associated with a newly imported 7PET strain, sublineage AFR15, from south Asia. All but 1 of the closest genomes were either collected locally and identified in Pakistan during June–December 2022 or detected within the framework of cholera surveillance in the United States or Australia (*8*). 

**Figure:**
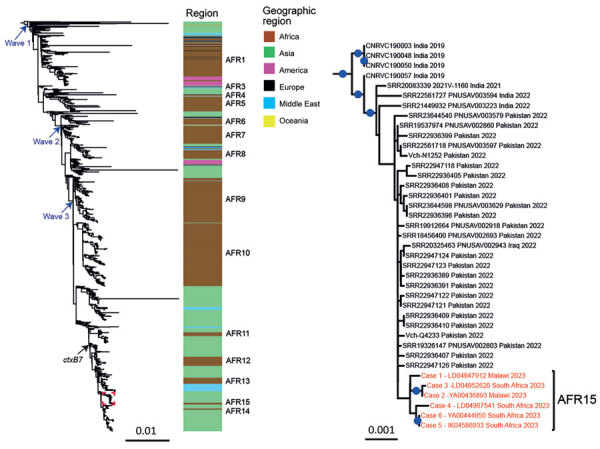
Maximum-likelihood phylogeny of *Vibrio cholerae* O1 El Tor isolates collected in South Africa, 2023, compared with 1,443 reference seventh pandemic *V. cholerae* El Tor (7PET) genomic sequences. A6 was used as the outgroup. The genomic waves and acquisition of the *ctxB7* allele are indicated. Color coding indicates the geographic origins of the isolates; sublineages previously introduced into Africa (AFR1, AFR3–AFR14) are shown at right. A magnification of the clade containing the 6 isolates from South Africa (red text) is shown at right. For each genome, name (or accession number), country where contamination occurred, and year of sample collection are shown at the tips of the tree. The 6 isolates collected in South Africa belong to a new 7PET wave 3 sublineage called AFR15. Blue dots indicate bootstrap values ≥90%. Scale bars indicate number of nucleotide substitutions per variable site.

In conclusion, we show that isolates from cases in South Africa, which have been linked to the 2022–2023 cholera outbreak in Malawi, belong to the seventh pandemic El Tor sublineage AFR15. Those cases did not result from resurgence of a strain previously circulating in any region of Africa but were caused by a cholera agent newly introduced into Africa from south Asia. This finding offers valuable information to all public health authorities in Africa. Genomic microbial surveillance and cross-border collaborations have a key role to play in identifying new cholera introductions, areas prone to cholera importation, and the main routes of cholera circulation. All of these elements are key to better understanding cholera epidemiology in Africa.

Appendix 1 Additional methods from study of imported cases of cholera in South Africa.

Appendix 2 Reference *Vibrio cholerae* isolates used in study of imported cases of cholera in South Africa.
