# Supplementary material for: Imported Cholera Cases, South Africa, 2023
Source: Emerg Infect Dis. 2023 Aug;29(8):1687–90. doi: 10.3201/eid2908.230750 (PMC10370840; doi:10.3201/eid2908.230750)
Supplement: Appendix 1 — Additional methods from study of imported cases of cholera in South Africa. [file 23-0750-Techapp-s1.pdf]

Article DOI: https://doi.org/10.3201/eid2908.230378
EID cannot ensure accessibility for supplementary materials supplied by authors. Readers who have difficulty accessing supplementary content should contact the authors for assistance.

Imported Cholera Cases, South Africa, 2023

Appendix 1

Materials and Methods

Phenotypic Characterization of Bacteria

The identification of *Vibrio cholerae* isolates was confirmed using standard phenotypic microbiological identification and serotyping techniques, briefly described as follows. Bacteria were sub-cultured onto 5% blood agar (Diagnostic Media Products, National Health Laboratory Service, South Africa) and Thiosulfate Citrate Bile Salts Sucrose Agar (Diagnostic Media Products) to check for purity of cultures. Bacteria were identified using the VITEK-2 COMPACT 15 automated microbial identification system (bioMérieux, Marcy-l'Étoile, France). Serogrouping and serotyping was determined by the slide agglutination method with polyvalent antisera and mono-specific Inaba and Ogawa antisera (Mast Group Ltd, Bootle, United Kingdom). Antimicrobial susceptibility testing was performed as follows.

The minimum inhibitory concentrations (MICs) of ampicillin, tetracycline, ciprofloxacin, and azithromycin were determined using the Etest method (bioMérieux). The Clinical and Laboratory Standards Institute (CLSI) interpretative criteria for antimicrobial susceptibility testing of *Vibrio* species (M45 document) were used when available (*1*). For antimicrobials not listed on this *Vibrio* species M45 document, the CLSI interpretative criteria for *Enterobacteriaceae*/*Salmonella* species (M100 document) were used (*2*).

Genomic DNA Isolation from Bacteria

Genomic DNA was isolated from bacteria using the Invitrogen PureLink Microbiome DNA Purification Kit (ThermoFisher Scientific, Carlsbad, California, USA), in accordance with manufacturer recommendations.

PCR for Toxin Detection and Biotyping

Real-time PCR was used to detect for the presence of cholera toxin (*ctxA* gene) (*3*). Conventional PCR and analysis of PCR products using agarose gel electrophoresis was used to detect the presence of allelic variants of the toxin co-regulated pilus (*tcpA* gene) which determined the biotype (classical or El Tor) of *V. cholerae* O1 (*4*).

Whole-Genome Sequencing (WGS)

We analyzed 6 *V. cholerae* O1 biotype El Tor isolates collected in South Africa by WGS (Appendix 2 Table 1; https://wwwnc.cdc.gov/EID/article/29/8/23-0750-App1.xlsx). WGS was performed using Illumina NextSeq (Illumina, San Diego, California, USA) next-generation sequencing technology; DNA libraries were prepared using a Nextera DNA Flex Library Preparation Kit (Illumina), followed by 2 × 150 bp paired-end sequencing runs with a mean coverage of 153-fold (range: 105-fold–257-fold). All reads were filtered with FqCleanER version 21.10 (https://gitlab.pasteur.fr/GIPhy/fqCleanER) with options -q 28 -l 70 to eliminate adaptor sequences and discard low-quality reads with phred scores <28 and length <70 bp (*5*).

Additional Genomic Data

Raw sequence files and assembled genomes from 1,437 7PET strains were downloaded from the ENA or GenBank and included in this study (Appendix 2 Table 3).

Genomic Sequence Analyses

The paired-end reads and draft or assembled genomes were mapped onto the reference genome of *Vibrio cholerae* O1 El Tor N16961, also known as A19 (GenBank accession numbers LT907989 and LT907990) with Snippy version 4.6.0/BWA v. 0.7.17 (https://github.com/tseemann/snippy). Single-nucleotide variants (SNVs) were called with Snippy version 4.6.0/Freebayes v. 1.3.2 (https://github.com/tseemann/snippy under the following constraints: mapping quality of 60, a minimum base quality of 13, a minimum read coverage of 4, and a 75% read concordance at a locus for a variant to be reported. An alignment of core genome SNVs was produced in Snippy for phylogeny inference.

Short reads were assembled with SPAdes v. 3.15.2 (*6*).

The various genetic markers were analyzed with BLAST v. 2.2.26 against reference sequences of the O1 *rfb* gene, *ctxB*, *wbeT*, and the whole locus of VSP-II, as previously described (*7*).

The presence and type of acquired antibiotic resistance genes (ARGs) or ARG-containing structures were determined with ResFinder v. 4.0.1 (https://cge.cbs.dtu.dk/services/ResFinder/), BLAST analysis against GI-15, Tn*7*, and SXT/R391 integrative and conjugative elements, and PlasmidFinder v. 2.1.1 (https://cge.cbs.dtu.dk/services/PlasmidFinder/). The presence of mutations in the genes encoding resistance to quinolones (*gyrA*, *parC*), resistance to nitrofurans (*VC_0715* and *VC_A0637*), or restoring susceptibility to polymyxin B (*vprA)* were investigated by manual analysis of the sequences assembled *de novo* with BLAST, as previously described (*7*,*8*).

Phylogenetic Analysis

Repetitive (insertion sequences and the TLC-RS1-CTX region) and recombinogenic (VSP-II) regions in the alignment were masked (*7*). Putative recombinogenic regions were detected and masked with Gubbins v. 3.2.0 (*9*). A maximum likelihood (ML) phylogenetic tree was built from an alignment of 10,679 chromosomal SNVs, with RAxML v. 8.2.12, under the GTR model with 200 bootstraps (*10*). This global tree was rooted on the A6 genome, and visualized with iTOL v. 5 (https://itol.embl.de) (*11*).

Data Availability

Short-read sequence data were submitted to the European Nucleotide Archive (ENA) (https://www.ebi.ac.uk/ena), under study accession number PRJEB39740 and their accession numbers are provided in Appendix 2 Table 3. WGS data were also uploaded to EnteroBase (https://enterobase.warwick.ac.uk/species/index/vibrio).

References

1. Clinical and Laboratory Standards Institute (CLSI). Methods for antimicrobial dilution and disk susceptibility testing of infrequently isolated or fastidious bacteria; approved guideline, third edition. CLSI document M45. Wayne (PA): The Institute; 2015.

2. Clinical and Laboratory Standards Institute (CLSI). Performance standards for antimicrobial susceptibility testing; twenty-seventh informational supplement. CLSI document M100-S27. Wayne (PA): The Institute; 2017.

3. Koskela KA, Matero P, Blatny JM, Fykse EM, Olsen JS, Nuotio LO, et al. A multiplatform real-time polymerase chain reaction detection assay for *Vibrio cholerae.* Diagn Microbiol Infect Dis. 2009;65:339–44. PubMed https://doi.org/10.1016/j.diagmicrobio.2009.07.009

4. Keasler SP, Hall RH. Detecting and biotyping *Vibrio cholerae* O1 with multiplex polymerase chain reaction. Lancet. 1993;341:1661. PubMed https://doi.org/10.1016/0140-6736(93)90792-F

5. Criscuolo A, Brisse S. AlienTrimmer: a tool to quickly and accurately trim off multiple short contaminant sequences from high-throughput sequencing reads. Genomics. 2013;102:500–6. PubMed https://doi.org/10.1016/j.ygeno.2013.07.011

6. Bankevich A, Nurk S, Antipov D, Gurevich AA, Dvorkin M, Kulikov AS, et al. SPAdes: a new genome assembly algorithm and its applications to single-cell sequencing. J Comput Biol. 2012;19:455–77. PubMed https://doi.org/10.1089/cmb.2012.0021

7. Weill FX, Domman D, Njamkepo E, Tarr C, Rauzier J, Fawal N, et al. Genomic history of the seventh pandemic of cholera in Africa. Science. 2017;358:785–9. PubMed https://doi.org/10.1126/science.aad5901

8. Weill FX, Domman D, Njamkepo E, Almesbahi AA, Naji M, Nasher SS, et al. Genomic insights into the 2016–2017 cholera epidemic in Yemen. Nature. 2019;565:230–3. PubMed https://doi.org/10.1038/s41586-018-0818-3

9. Croucher NJ, Page AJ, Connor TR, Delaney AJ, Keane JA, Bentley SD, et al. Rapid phylogenetic analysis of large samples of recombinant bacterial whole genome sequences using Gubbins. Nucleic Acids Res. 2015;43:e15. PubMed https://doi.org/10.1093/nar/gku1196

10. Stamatakis A. RAxML-VI-HPC: maximum likelihood-based phylogenetic analyses with thousands of taxa and mixed models. Bioinformatics. 2006;22:2688–90. PubMed https://doi.org/10.1093/bioinformatics/btl446

11. Letunic I, Bork P. Interactive Tree Of Life (iTOL) v4: recent updates and new developments. Nucleic Acids Res. 2019;47(W1):W256–9. PubMed https://doi.org/10.1093/nar/gkz239
